# A Qualitative Study on Noncommunicable Diseases in Waste Pickers in Brazil

**DOI:** 10.5696/2156-9614-11.30.210603

**Published:** 2021-05-28

**Authors:** Tara Rava Zolnikov, Vanessa Cruvinel, Paola Lopez, Farid Pezeshkian, Lakeithia Stoves-Tucker, Dayani Galato, Carla Pintas Marques

**Affiliations:** 1 Department of Community Health, National University, San Diego, CA; 2 Department of Public Health, University of Brasilia, Brasilia, Brazil

**Keywords:** qualitative, waste pickers, hypertension, diabetes mellitus, solid waste segregators, social conditions

## Abstract

**Background.:**

Noncommunicable chronic diseases are associated with multiple risks factors and negative outcomes that are long-lasting and difficult to treat. Some populations may be at greater risk because of their socioeconomic status, lack of healthcare, environment, and poor work and living conditions. Informal waste pickers may experience higher levels of chronic diseases and often do not have access to care to manage symptoms.

**Objectives.:**

The aim of the present study was to understand the prevalence of chronic diseases in waste pickers, along with perceived associated risks and available treatments.

**Methods.:**

A qualitative study was conducted, using interviews with 24 waste pickers who worked at *Estrutural* dumpsite in Brasilia, Brazil which was historically the second largest open-air dumpsite in the world.

**Results.:**

Participants believed their commonly experienced chronic diseases were a result of working in the open-air dumpsite. Chronic diseases commonly noted in the interviews included hypertension, chronic pain, respiratory disease, diabetes, and kidney problems. Participants discussed self-medication or prescribed medication used to treat their conditions. Most participants had varying beliefs regarding prevention strategies to reduce disease; some ideas for prevention focused on religion, fate, and God when discussing outcomes related to illnesses. When answering questions regarding ideal working conditions to help prevent diseases, participants responded by expressing a desire for protective gear (e.g. PPE) which could help mitigate hazards associated with the dump.

**Conclusions.:**

Recyclable collectors were aware of occupational hazards to which they were exposed and associated noncommunicable chronic diseases but lacked education on the importance of preventive measures and access to healthcare services. The findings of the present study confirm the need to strengthen intersectoral actions to protect and uphold the health rights of this vulnerable population.

**Participant Consent.:**

Obtained

**Ethics Approval.:**

This study was approved by the Research and Ethics Committee of the Health School of Brasília University under Opinion n. 1.517.670/2016.

**Competing Interests.:**

The authors declare no competing financial interests.

## Introduction

Brazil creates 79 million pounds of waste per year and has the fifth highest contribution of trash and waste products globally.^1^ Until 2018, Brazil was home to the second largest open-air dump in the world, covering over 300 acres of land, until officially closed by the government.^2^,^3^ Since then, Brazil has continued to operate smaller open-air dumps throughout the country, mainly in poorer neighborhoods and communities. Approximately 3000 dumpsites remain in operation, impacting the quality of life of 77 million Brazilians through contaminated air, soil, groundwater, and superficial waters.^3^,^4^ An open-air dump is a site where solid waste is disposed of in a manner that does not protect the environment or the residents of the area.^5^ Piles of garbage accumulate and expose nearby residential and working populations (e.g., waste pickers) to various hazards (e.g., physical, biological, etc.).^5^

Waste pickers are exposed to multiple hazards^3^,^5^; they work by sorting through garbage to find recyclable material of monetary value.^3^,^5^ Unfortunately, this informal occupation continues to have a presence despite dump closures in Brazil.^5^ To date, there are an estimated 200 000 to 800 000 waste pickers in Brazil, with the majority working alone and informally.^1^,^3^,^5^ This suggests that there is a significant population that is exposed to the effects of open-air dumps. These adverse health effects can include physical (e.g., slips, trips, falls), biological (e.g., hepatitis B and C), chemical (e.g., burns), ergonomic (e.g., heat stress), and social (e.g., poor access to healthcare, clean water, etc.) effects.^3^,^5^,^6^,^7^ Unfortunately, waste pickers frequently do not receive healthcare treatment for these exposures and adverse health effects that they experience.^8^ Access to healthcare in Brazil may be even more difficult due to social disparities in vulnerable populations.

While Brazil has made recent strides in improving access to healthcare, there are still health disparities and issues within the system. Brazil currently operates under a universal health care model that also allows citizens to purchase private insurance to supplement existing coverage.^8^ The substantial health disparities in Brazil are highlighted by the fact that 45% of the adult population has reported having at least one noncommunicable disease.^10^ Noncommunicable (chronic) diseases (NCDs) can affect a person's quality of life, impact economic stability, and reduce the ability to live independently. Brazil continues to treat noncommunicable diseases as a priority in its healthcare campaigns, although 72% of all deaths in the area are attributable to these chronic disorders.^11^ Unfortunately, most of those deaths occur in the poorest segment of the population, who are often also the most vulnerable due to lack of resources, increased risks, etc.^11^

Vulnerable populations could be more situationally affected by the disease because of poverty and poor working conditions, such as experienced by waste pickers. The objective of the present study was to understand the relationship between noncommunicable chronic diseases in Brazilians working as waste pickers. Solutions to improve these disease outcomes and improve occupational safety among workers were also reviewed. Ultimately, this type of information could highlight areas of improvement to decrease noncommunicable diseases in at-risk populations.

**Abbreviations**
*NCD*: Noncommunicable disease

## Methods

A descriptive phenomenological qualitative study was used to explore the experiences of waste pickers and chronic disease. The purpose of using this type of study was to review and gather multiple realities, perceptions, perspectives, and experiences.^12^,^13^,^14^ A phenomenological study delves into the experiences of each participant to understand meanings instead of making inferences of a situation, which can allow a comprehensive review of the phenomenon being explored.^15^,^16^,^17^,^18^ Phenomenological research methods require the researcher to set aside preconceived notions and biases, while still connecting to the participant to collect data.^19^,^20^ These aspects can encourage validity while gathering data, although it should be mentioned that researcher bias could still exist. Validity and reliability of qualitative research is based on the credibility, trustworthiness, and authenticity of the information collected during the study.^18^ The health belief model was integrated in this research from a theoretical perspective to explain noncommunicable diseases that could be experienced by waste pickers. The health belief model refers to a person's belief or perception of associated risk in acquiring a disease.^21^

### Sampling

Data were collected from waste pickers located in Brasilia, Brazil who worked in the largest open-air dump in Latin America. Data were collected between 2019 and 2020. Inclusion criteria included people who identified and worked as waste pickers in the dumpsite who were above the age of 18 years old in order to be inclusive of waste pickers of various ages, genders, and possible health conditions. Convenience sampling was used to gather eligible participants. Qualitative methodology focuses on gathering participants until the point of data saturation, so participants were recruited until repetitive information was described amongst the selected population. Participants were recruited by cell phone to participate in the study; this was part of a protocol that also used a knowledge exchange to discuss and empower participants concerning the most common hazards and outcomes detected by an epidemiological survey conducted with this group before the dumpsite closure.^3^ Areas of exploration included waterborne diseases, noncommunicable diseases, communicable diseases, sexually transmitted infections (STIs), accidents, and occupational hazards. In addition to the survey, waste pickers were invited to participate in this qualitative study to triangulate data. Prior to interviews, all participants could ask questions about the study. Individuals with low or no literacy had the consent form read to them and all of them gave informed consent.

### Ethics Approval

The present study was approved by an institutional review board of the University of Brasilia under protocol number 55754216.5.0000.5553. This study complied with all ethics committee rules for conducting and collecting human participant data. All participants were informed that their decision to participate in the study was voluntary, would not affect current or future care, and that participation could be terminated at any time without penalty. The confidentiality of the participants and associated health conditions were protected.

### Data collection instruments

The research team used a self-reported health questionnaire which was divided into three categories: work accidents, sexually transmitted diseases, and noncommunicable chronic diseases *(Supplemental Material).* This study focused specifically on NCD using topics and variables to uncover information related to these diseases *(Table 1).*

**Table 1:** Questionnaire Topics and Variables

	**Variables**
**Socio-demographic variables**	Gender; age group; marital status; number of children
**Chronic disease (CD)**	Diabetes, hypertension; kidney problems; chronic pain; respiratory disease; others
**Health access**	Access to treatment; last visit to a doctor; use of continuous medication; self medication
**Perception of risk**	Knowledge to prevent CD; relationship between CD and work; perception of work conditions

### Interview process

Semi-structured interviews were used to create a flexible conversation with the participants regarding their experiences.^22^ This process encouraged detailed and lengthy descriptions from waste pickers on their experiences with chronic diseases. The interviews were audio recorded and data from the study was then transcribed, analyzed, and coded using the methods of Christians and Carey.^23^

### Data analysis

The validity of the study was upheld by ensuring that the conclusion of the study accurately represented the phenomena being studied.^24^ Trustworthiness for validity of a study involves credibility, transferability, and confirmability.^25^ Credibility involves persistent observation, peer debriefing, and member checking^26^; these actions occurred in this study, as the research team worked together to confirm questions, themes, and conclusions. Transferability was sought by providing evidence (e.g., triangulation of data) that the study could be applied to other contexts; triangulation of the themes through various data sources was used to ensure data consistency by the participants in this study and in general.^24^ Confirmability was achieved when the research team checked and rechecked data, while searching for outcomes that were related to health and access to healthcare.

## Results

There were 16 females and 8 males (n = 24) included in this qualitative study. The average age was 53 years old; participants had a range of marital status and amount of dependents (e.g. children), and education levels were low. In relation to self-reported chronic diseases, women appeared to be more affected than men; most of the women were over 45 years old, single or divorced, and with low levels of education *(Table 2).* All participant data has been deidentified and coded as P for ‘participant' and the subsequent number in which they were interviewed.

**Table 2:** Sociodemographic Variables in Relation to Chronic Disease

		**Chronic Disease N (%)**	**Chronic Disease N (%)**	**Total N (%)**
		**YES**	**NO**	
**Gender**	Female	15 (93.7%)	1 (62.5%)	16 (66.7%)
	Male	3 (37.5%)	5 (62.5%)	8 (33.3%)
**Age group (years)**	35 and under	3 (100.0%)	0 (0.0%)	3 (13.1%)
	36–45	2 (100.0%)	0 (0.0%)	2 (8.7%)
	over 45	12 (66.6%)	6 (33.4%)	18 (78.2%)
**Marital status**	Single/divorced	9 (75.0%)	3 (25.0%)	12 (70.6%)
	Married/partner	2 (50.0%)	2 (50.0%)	4 (29.4%)
**Schooling (years)**	0	4 (100.0%)	0 (0.0%)	4 (19.5%)
	1–4	4 (50.0%)	4 (50.0%)	8 (38.0%)
	5–8	5 (71.4%)	2 (28.6%)	7 (33.0%)
	More than 8	2 (100.0%)	0 (0.0%)	2 (9.50%)
**Number of children**	0	0	0	0
	1–3	8 (72.7%)	3 (27.3%)	11 (50.0%)
	4 and more	8 (72.7%)	3 (27.3%)	11 (50.0%)

All the participants were waste pickers and 23 had actively worked at the waste dump site before the dumpsite closure and were currently working independently in the street waiting for a spot in the sorting plants and one person was no longer able to work due to health complications caused by diabetes. The information obtained from the interviews was indicative of a perceived relationship between the development and/or worsening of noncommunicable chronic diseases and occupation as a waste-picker. The chronic diseases that were commonly noted throughout the interviews included hypertension (45.8%), chronic pain (29.1%), respiratory disease (20.8%), diabetes (12.5%) and kidney problems (8.3%). Of the 18 workers who reported NCDs, nine (9) (50.0%) had been diagnosed by a physician and were monitored by the healthcare system, and nine (9) (50.0%) had not been diagnosed nor were they currently receiving any health care treatment. Among those who had reported experiencing NCD, the majority were women. Women were more informed than men on how to prevent NCD and were more aware about the relationship between NCD and work. Only four workers described practicing self-medication *(Table 3).*

**Table 3:** Healthcare Access, Disease Prevention Knowledge, Perception of the Relationship Between Occupation and Chronic Disease, and Use of Self-medication among Waste Pickers Experiencing Chronic Disease

**Health conditions**		**Men N (%)**	**Women N (%)**	**Total N**
**Chronic disease**	Diabetes	0	3 (100.0%)	3
	Hypertension	2 (18.2%)	9 (81.8%)	11
	Kidney problems	0	2 (100.0%)	2
	Chronic pain	2 (28.6%)	5 (71.4%)	7
	Respiratory disease	1 (205)	4 (80.0%)	5
**Healthcare access**	Yes	0	9 (100.0%)	9
	No	3 (33.3%)	6 (66.7%)	9
**Knowledge of chronic disease prevention**	Yes	1 (20.0%)	4 (80.0%)	5
	No	0	0	0
	Did not answer	2 (15.4%)	11 (84.6%)	13
**Perception of relationship between chronic disease and work**	Yes	1 (16.7%)	5 (83.3%)	6
	No	1 (100.0%)	0	1
	Did not answer	2 (19.0%)	9 (81.0%)	11
**Self-medication**	Yes	1 (25.0%)	3 (75.0%)	4
	No	1 (100.0%)	0	1
	Did not answer	2 (15.4%)	11(84.6%)	13

### Working environment

Worksite conditions for waste pickers are significantly poor; waste pickers are often exposed to adverse weather conditions, contaminated water, and hazardous terrain that can contribute to slips, trips, and falls. The development of chronic illnesses has been credited to working conditions in the “shed” with participants stating the following:

“…you eat very badly, you are very exposed to the sun, then there are problems with the views…I was blind [at times], I was in the very hot sun and then came the rain… and as you have nowhere to go, [you can] catch something very [harmful]. [It] is very difficult there” (P5).

Participants also noted conditions that were a direct result of hazards and poor work conditions at the waste site; this situation led to broken bones, renal disease, and spinal problems, such as pain and disc herniation. One participant with chronic back pain stated that his spinal condition developed “because I carried a lot of weight” (P18). These working conditions contributed to disease prolongment in waste pickers. Another participant believed that she may have developed multiple health conditions specifically attributed to working as a waste picker. “I think it was in the dump, [I] started [having] high blood pressure and then came kidney problems… and [eventually] found diabetes by treating the kidneys (P21)”.

These adverse health conditions often affected their ability to work as a waste picker, “it can get in the way” (P1). The working environment and waste pickers' health problems also affected waste pickers emotionally. “Sometimes it gets in the way, it makes me very tired, discouraged, and it gets in the way a little” (P12).

### Diseases and treatment

Participants in the present study were verbally asked if they had experienced any noncommunicable chronic diseases, which did not need to be confirmed by a physician. Some participants did not report any chronic conditions, with some participants reporting that they could not confirm the presence or absence of chronic disease, since they had never been tested. With regard to chronic symptoms, some participants reported experiencing constant pain and indicated that they had experienced a number of conditions or ailments, including diabetes, chronic pain, high blood pressure, inflammation, stroke, kidney disease, spinal problems, headache, prostate problems, sinusitis, allergic rhinitis, polycystic ovary syndrome, and edema. Participants stated that these diseases were related, caused or aggravated by their job activities. “Of course [I have a chronic disease because of working in the dump]. I have a cholesterol problem, I have high blood pressure… I take insulin in the morning, in the afternoon… all because of the dump. I am swollen [and in pain and only] take medicine to detach [and relieve myself from the pain]” (P16). Other participants reaffirmed that most of their diseases and ailments occurred because of working in the dump.

My feet are all swollen… It turned out that I have diabetes… [my] veins are all clogged, I have a] heart problem, everything… I think I got it from working in the dump; I think I got [all] these health problems [from working in the dump]” (P2).

Participants who were diagnosed with chronic ailments complained that while they took medication for their conditions, visits to the health clinic were not helpful.

“Whenever I go there, [I just] return… you go there and sometimes [you don't receive any treatment or care and] people do not attend to you. So, my legs… [they] say it is a disease that will not heal, [so, they] pass a dipyrone (a pain reliever) and send [me] home” (P5).

Another participant described the lack of exams and care provided at the local health center and the need to rely on family for help. “I should have an exam every four months, but [instead], I can only change the medicament prescription because there are no doctors [to give me an exam]. My daughter is doing my pressure monitoring, she is a nurse” (P19). Participants reported treatments that included visits to the health center, oral medications, home remedies, injections, and special diets, although sometimes participants were unable to continue with this care. “My husband and I are unemployed… I'm out of tablets and we have no financial resources to buy more. And because of this, I'm not taking the tablets anymore” (P1).

When they were asked about self-medication, some participants reported this habit, especially for chronic or acute pain, while other participants reported they took prescribed medication at the health care center.

### Prevention

Participants were asked about their knowledge of disease prevention and actions they would take in order to prevent illnesses and noncommunicable chronic ailments. Many participants reported they knew how to prevent these diseases. However, some participants focused specifically on their religious beliefs and how they would “just ask God” (P5), while others stated they would not take preventive actions. Some participants stated they would try to do both. “It [takes] more prayer *and* a healthier diet, because we do not get rid of the problem… [only] God [can really] save us” (P5). Another participant described ways they prevented chronic diseases in their own life. “I avoid certain kinds of foods that can cause these diseases. If I don't have these diseases or if I do, at least I avoid these diseases from doing greater damage [by avoiding] eating fatty foods, lots of sweets… things like that” (P6).

### Solutions

When answering questions regarding ideal working conditions to help prevent diseases, participants responded by expressing a desire for protective gear (PPE), such as gloves and face masks. Participants believed that this type of gear would prevent them and other workers from directly inhaling the air in the dump or shed as well. Workers ultimately wanted “a healthy environment… clean more or less [of] that” (P11). Another participant stated that ideal working conditions would involve overall improved working conditions. “[We need] a more suitable place with sanitation, because lately, [we don't have anything and it] is very dirty. [We have] no people to help [us] and inform people [about what is going on here]. [Someone needs] to teach people and [improve the] lack [of] awareness of [waste pickers and their conditions]” (P14). Other common responses included having retirement benefits and resignation to the fact that the conditions in the shed are unlikely to change. Finally, participants expressed their desire to change jobs, stating “my dream is to sweep the streets” (P3). Participants believed that these solutions could create better working conditions and could help prevent chronic diseases.

## Discussion

Qualitative research can provide a baseline of information through exploratory studies, which can be used as a springboard for further quantitative confirmatory studies. As such, although some reports were undiagnosed and not confirmed by physicians, it is not surprising that chronic diseases affected waste pickers. The results of this study confirmed the significant effects that these types of diseases can have on the lives of waste pickers. Waste picking is already a difficult job, as workers are exposed to various environmental conditions and hazards, and the addition of chronic diseases and pain worsens the situation for these workers. A previous study confirmed that for noncommunicable disease-related health outcomes in waste pickers in a sample size of 268, the prevalence of hypertension was around 33%, and approximately 51% and 26% of workers were overweight and obese, respectively.^27^ This study also reaffirmed that approximately 15% had never accessed healthcare and despite weight-related issues, 45% and 61% had never had a glucose or triglyceride test, respectively.^27^

Currently, chronic diseases account for 75% of all deaths in low-and middle-income countries.^28^ This problem is compounded by barriers to receiving proper care and treatment. Although many waste sites have been closed throughout Brazil, many waste pickers continue to work and are exposed to hazards while earning low wages.^5^ The results of this study also confirmed that many participants did not know if they had any noncommunicable diseases, since they had never formally been tested. Many participants believed that their conditions occurred as a result of working in the dumpsites, such as spinal problems, chronic headaches and pain, allergic rhinitis, and sinusitis. Other noncommunicable diseases such as diabetes, kidney disease, and prostate problems were also discussed. Many of these diseases and outcomes can be managed with proper medical interventions.

The environmental, occupational, and social hazards waste pickers are exposed to increase their chances of illness. In the present study, most of the participants were elderly women, single or divorced, with low education levels, many children to raise, poor healthcare access and without social security, living and working in poor conditions and consequently were also likely suffering with a high prevalence of noncommunicable diseases. According to primary care policy in Brazil, all people with noncommunicable diseases should be monitored by a Family Health Strategy Team and receive all medications for free.^8^ In 2011, Brazil developed the Strategic Action Plan for Coping with Chronic Non-Communicable Diseases, with the objective of promoting the development and implementation of effective, integrated, sustainable and evidence-based public policies for prevention, control, and care of NCDs and their risk factors.^29^ Unfortunately, this program focuses on home visits and because waste pickers are away from home working in the dumpsite, they are not covered by this prevention program. These types of efforts and solutions need to be expanded upon to include this vulnerable population [8]. Studies have shown that to improve access to primary health care, there is a need for increased investments, expanding the coverage of health teams, reducing their caseloads, increased medical training in family and community medicine, expanding nursing clinics, diversifying the routes of communication with users and increased appointment flexibility.^30^ Waste pickers are part of an informal industry, and do not have access to social benefits, including insurance^31^,^32^ making it difficult for them to seek and receive the care they need.^8^ Moreover, lack of education is a barrier to assessing risks and poverty (e.g., inability to pay for services) can also contribute to disease proliferation.^31^,^32^ Interestingly, a novel aspect that arose from this research concerned religious beliefs, as most participants believed that prayer could assist in healing. For example, when participants were asked about disease prevention, many participants mentioned how they would “just ask God [for help],” suggesting that they would be unlikely to take concrete actions to prevent their chronic disease. This information suggests that education, health insurance, and access to healthcare are primary components in preventing and managing chronic diseases for waste pickers.

In more of a macro-lens, waste picking needs to be recognized as an occupation to remove barriers, like poor access to healthcare and associated health effects, that are often produced within informal industries. National and local governments should collaborate and strengthen programs, cooperatives, and initiatives that can decrease the risk of accidents and disease and ultimately, improve health outcomes in waste pickers.^33^ Additionally, education on chronic disease, including symptoms, treatment, and prevention may provide important information for waste pickers. While current research supports strengthening national healthcare systems^34^,^35^,^36^, smaller-scale interventions for management of chronic diseases may be used to deliver information that could improve outcomes in waste pickers. For example, in two countries in Africa, chronic disease education was provided by churches.^37^ This could be a possible avenue to disseminate information, as most participants in the study had strong religious affiliations. Another program found positive outcomes by using community health workers to bridge the gap between healthcare services and vulnerable populations^38^; this study highlighted the value of community health workers being able to enhance services and information, especially regarding chronic diseases.^38^ Finally, programs focused on self-management education can improve health-promoting behaviors and have been utilized for many chronic conditions, like arthritis, asthma, diabetes, and hypertension.^39^ This type of program could prove useful in addressing chronic diseases in waste pickers as well.

Finally, it should be further noted that the sample in the present study was included in a previous quantitative study, wherein 1 025 waste pickers underwent tests and participated in interviews.^3^ Most participants were women between 36–45 years old and the mean time working as a waste picker was 15 years. According to the anthropometric measures, 32.6% were overweight and 21.1% were obese. The most common NCDs reported in this large quantitative study were osteomuscular disorders (78.7%), hypertension (24.2%), bronchitis (14.3%), and diabetes (10.1%).^3^ The results of the present qualitative study found participant disease descriptions to be more linked to hypertension, respiratory disease, and diabetes, perhaps due to the fact that most of the 24 waste pickers that were interviewed were older (over 50 years old). One concern about this lack of triangulation of results is the underestimation of kidney problems and diabetes reported by participants in comparison to blood exam results in the previous quantitative study of Cruvinel *et al.*^3^, in which a high percentage of creatinine (54.06%) and glucose (35.5%) results were outside reference limits. These results highlight the importance of following up with workers to educate them on diseases, symptoms, and prevention.

Finally, participants also reported the practice of self-medication. This practice is recommended by the World Health Organization (WHO) for patients without chronic disease^40^ [40], but this was not the case in the present study. Waste pickers have been reported to obtain medicines from the trash, as observed by Ramos *et al*.^41^ This practice self-medication could cause other problems for this group, such as renal diseases.^41^

### Limitations

The most important limitation of the present study is that chronic diseases reported by waste pickers were often not verified through physician diagnosis. However, we believed it was important to gather information on chronic diseases in this population, which faces many barriers to accessing healthcare services.

This qualitative research gathered data on waste pickers in Brasilia, Brazil. The present results may not be transferable to waste pickers worldwide. Other factors may affect waste pickers in other areas of the world, with differences in diet (e.g., meat, sugary foods, etc.), activity levels, smoking, alcohol, and obesity. Finally, the self-reported nature of data collection is another major limitation.

## Conclusions

Recyclable collectors were aware of occupational hazards to which they were exposed which often contributed to associated chronic noncommunicable diseases. However, they lacked education on the importance of preventive measures and access to healthcare treatment. There is a need to strengthen intersectoral actions for the protection and defense of the health rights of this vulnerable population. Developing local mechanisms to reduce health inequities in urban cities globally can improve health outcomes. There is a need for effective service management (e.g., waste management, recyclable materials collection) as well. Finally, future research should focus on more quantitatively assessing the relationship between occupational exposures and chronic diseases in waste pickers to determine the link between exposures and health outcomes.
