# Supplementary material for: A Qualitative Study on Noncommunicable Diseases in Waste Pickers in Brazil
Source: J Health Pollut. 2021 May 28;11(30):210603. doi: 10.5696/2156-9614-11.30.210603 (PMC8276723; doi:10.5696/2156-9614-11.30.210603)
Supplement: Supplementary file 1 [file Zolnikov_Supplemental_Material.docx]

**Supplemental Material**

**Noncommunicable Chronic Disease Interview**

**Waste Picker ID#:
Name:**
**Age:**
**Marital status:**  **Schooling:**  **Nº of children:**

**Do you work in the shed?**

**Do you have any noncommunicable chronic diseases?**

**If so, which?** *Examples: (hypertension, diabetes, kidney problems, chronic pain, respiratory disease, others)*

**What are your barriers to accessing the health center?**

**How often do you go to the emergency room?**

**Do you know anyone with a noncommunicable chronic disease?**

**Do you know how to prevent these diseases?**

**Is there any relationship between noncommunicable chronic disease and your occupation?**

**How could your working conditions be improved?**

**Do you have any other health problems?**

**Do you experience chronic pain?** **If so, how do you treat your pain?**

**Do you take any medicine? If so, which ones?**

**When was the last time you went to the doctor?**
